# Taking high-stakes venture to make ends meet? Determinants and impacts of international migration of Ethiopians to the Middle East

**DOI:** 10.1186/s40878-023-00338-z

**Published:** 2023-05-01

**Authors:** Beneberu A. Wondimagegnhu, Lemlem Fantahun

**Affiliations:** 1 Economic Policy Studies Sector, Ethiopian Policy Studies Institute, P.O. Box 2479, Addis Ababa, Ethiopia; 2 Department of Rural Development and Agricultural Extension, Woldiya University, Woldiya, Ethiopia

**Keywords:** International migration, Determinants, Impact, Income, Propensity score matching, Ethiopia

## Abstract

Ethiopia is one of the major origins for international migrants to the Middle East in Africa regardless of the risks and the abuses that migrants face. The study aims to analyse the determinants of international migration of Ethiopians to the Middle East and its impact on the income of households staying behind particularly in the Dessie Zuria district of the Amhara region in Ethiopia. Data were randomly collected from 346 households and analysed using descriptive statistics, logit regression, and Propensity Score Matching (PSM) models. The logit regression analysis indicates that bigger family size, network with migrants/returnees, and the presence of peer/family pressure influence the probability of international migration positively. On the contrary, being a male household head, ownership of larger farmland and livestock, and participation in additional non-farm activities determine international migration negatively. The findings from the PSM model revealed that international migration increased the annual income of migrant-sending households by 13,079.51 ETB per year over non-migrant sending households. However, the benefits enjoyed by the families staying behind have been at the expense of migrants, whose income is hard-earned and they often take a risky route to reach the destination regions. The findings call for an integrated policy approach to control population pressure that depletes the key financial and physical assets of households in the origin and thus forces individuals to look for alternative livelihood strategies such as migration. Location-specific policy interventions are needed to create non-farm and alternative livelihoods, improve agricultural productivity, and access information to reduce exaggerated and misleading information about the destination areas.

## Introduction

Migration is a multifaceted issue affected by several factors such as social, environmental, economic, and geographic variables.^1^ It is a large concern for policymakers as the flows of the population can significantly affect local politics, and social, economic, and ecological structures for both sending and receiving countries (Gemecho & Goshu, 2018).

The IOM report in 2020 on migration indicates that the number of international migrants has grown exponentially in the last ten years. The number of migrants shows an increase from 222 million in 2010 to 244 million in the year 2015, and 272 million in 2019 accounting for 3.5% of the world's total population. Out of these migrants, 48% and 52% were females and males respectively. Moreover, about three fourth of all international migrants are in a working-age group, i.e. 20–64 years. India is the largest origin for international migrants with 17.5 million migrants, followed by Mexico (11.8 million migrants) and China (10.7 million migrants). The United States of America is the top destination country for 51 million migrants. It is followed by Saudi Arabia which accounts for 13 million immigrants in 2019implying that 38% of Saudi Arabia's population are immigrants (IOM, 2020a).

According to the 2020 world migration report, international remittance has increased to 689 billion USD in 2018. India is the top remittance recipient country from other countries (i.e. 78.6 billion USD) followed by China and Mexico with 67.4 and 35.7 billion USD, respectively. United States is the top remittance-sending country (i.e. USD 68 billion) followed by the United Arab Emirates and Saudi Arabia, each accounting for 44.4 and 36.1 billion USD(IOM, 2020a). In globalization, improved and sophisticated means of transportation and communication play a key role in facilitating the interaction of people around the world that initiate people's mobility. As a result, the rate of migration has been increasing both at national and international levels (Meron, 2017).

Various factors determine people’s inherent motive to migrate from one place to another. Researchers agree that migration may be driven by both “push factors” in the origin area such as social inequality and poverty, and “pull factors” in the destination area such as better economic opportunities and social safety (IFAD, 2018).

Patterns of migration have been varying over time across countries and regions. Migration is a fundamental component of structural transformation in developing countries. Migration has a great contribution to all aspects of economic and social development and it is also used as a key instrument for achieving Sustainable Development Goals (SDGs). It is also used as an effective poverty reduction tool for migrants, migrant-sending families, and the communities as a whole (Foresti & Hagen-Zanker, 2018).

Ethiopia is one of the countries in Africa with the largest number of refugees and at the same time one of the major labour-sending countries in the world (ILO, 2017). Although the economy of Ethiopia shows growth in the last two decades, it has not been accompanied by a significant reduction in poverty and unemployment particularly for the growing youth (ILO, 2017). As a result, many Ethiopians still consider out-migration as the only available way to improve their living standards (De Regt &Tafesse, 2016). The country has a population of almost 120 million, of which 39% are below the age of 35. According to the report from the Ethiopian Ministry of Labour and Social Affairs (MOLSA), there were approximately 11 million youth job-seekers as of mid-2019 and two million more youth join the labour force every year (Zerihun, 2019). There could be multiple reasons why migrants move such as joining family, studying, escaping violence and conflicts, or seeking jobs (Stefanie & Yannis, 2019). International migration for employment and associated pressures will continue to grow in volume and complexity due to different reasons like globalization, supply, and demand of the labour market as well as other factors.

As the population rate in Ethiopia increases, the demand for jobs has continued to rise. However, not more than one million jobs are created by the government per year. The economy is not also generating adequate jobs to address Ethiopia’s increasing demand, which partly explains the country’s high unemployment rates. As a result, several Ethiopians continue to migrate internationally looking for employment opportunities (Ayalew et al., 2020).

Ethiopia has a long history of labour migration to the Middle East as compared to other East African countries mainly for low-skilled and unskilled jobs. At least for the last two decades, Ethiopian women have been migrating to the Middle East and Gulf countries (Demissie, 2017). According to the report from African Union Commission and JLMP partners (2017), the majority of Ethiopian workers in the Middle East are engaged in domestic work like nursery and caretaking of elders, while others work as daily labourers mostly in construction sites, and some of them are engaged in agricultural activities, especially animal husbandry and low skill construction activities. For many Ethiopians, international migration especially to the middle-east countries is perceived as a primary solution to an impoverished living situation. Migration is still a livelihood choice that many Ethiopians prefer regardless of the awareness of the risks and the abuses that happen to them. The success of migrants in Arab countries has been regarded as a matter of luck. Those fortunate enough would change their lives and that of their families while others lost their lives along the way and others are left stranded amid bewilderedness. The majority of Ethiopian migrants use informal routes including travelling overland through Somalia and crossing to the Gulf countries by boat to reach the Middle East. Besides, some migrants travel to the Middle East on tourist visas and become irregular by overstaying their visas. Both in the course of their journeys and upon arrival to the destination countries, Ethiopian migrants particularly those who are irregular become vulnerable to different forms of exploitation (Demissie, 2017). Many Ethiopian irregular migrants are recruited by local brokers and some of them are relatives, friends, neighbours, and returnees. In such situations, informal brokers offer potential migrants false promises of good financial revenues that will enable the migrants and their families to escape from poverty. As a result, irregular migrants neither do receive relevant information before their departure nor are they allowed to discuss the terms and conditions of their jobs with their employers or recruiters. ILO (2017) reported that more than 30% of Ethiopian migrants had no information about the nature of their jobs, and 54% had no information about their employers before their travel to the Middle East.

Although migration has contributed to the improvement of livelihoods, many of the young Ethiopians who migrate to the Middle East are not aware of the risks of the journey. They experience hunger, dehydration, and contract water-borne and gastrointestinal diseases with the possibility of being abused, exploited, and killed. More than one million Ethiopian migrant workers are found in the Middle East. Out of this number, many of them are undocumented and currently, they have been stranded amid the outbreak of the COVID-19 pandemic. Due to the pandemic and its subsequent impact on the economy of Lebanon, thousands of irregular and undocumented migrants have already lost their jobs. As a result, many of them have begun returning to Ethiopia (IOM, 2020b, 2020c).

A study conducted by Shishay (2019) assessed the drivers and life experiences of migrants in Ethiopia. The literature fairly studies the driving forces or determinants of international migration. However, the study is conducted in two different districts of the Tigray region, and thus the findings of the study could not be generalized to other regions of Ethiopia as the economic and socio-cultural background of migrants were not addressed. In addition, this study did not include the impact of migration. Another study conducted by Melaku (2018) assessed the causes and consequences of migration to Arab countries but does not include the economic impact of migration particularly on sending households. The other study on international migration by Giorgis and Molla (2013) explores the impact of international remittance on poverty, household consumption, and investment in urban Ethiopia. This study fairly addressed the impact of international migration through remittance on poverty but the key determinants that influence migrants’ decision to migrate were not included. Similarly, Kerime and Degefa (2016) have studied the contribution of remittance to the improvement of rural households’ livelihoods in TehuledereWoreda, Northeastern Ethiopia. In this literature, the impact of international migration through remittance was fairly addressed but it did not include the determinant factors of international migration. Several studies such as Shishay (2019), Mulugeta and Makonnen (2017), Yohannes (2018), Girmachew (2018), Mesfin and Emirie (2018), Atnafu et al. (2014), Abel (2019), and Aragie and Zerihun (2019) have studied international migration in Ethiopia. However, they focused either on the determinants of international migration or the impacts. In addition, the studies that particularly focused on the impacts of international migration on the livelihood of migrant-sending families did not include a comparative assessment with those who do not send migrants. This study contributes to filling the existing literature gap by simultaneously assessing the determinants and impacts of international migration. In addition, the study contributes to location-specific and household-level studies on migration and includes a comparative assessment with non-migrant sending families so that the change in income of households as a result of international migration could be easily measured at a micro-level. This study contributes to designing appropriate local migration policies and strategies so that the positive impacts of international migration are maximized and the negative outcomes are lessened. Therefore, this study analyzes the determinants of international labor migration and its impact on households’ income particularly taking the case of north-central Ethiopia.

## Materials and methods

### Location of the study area

**Fig. 1:**
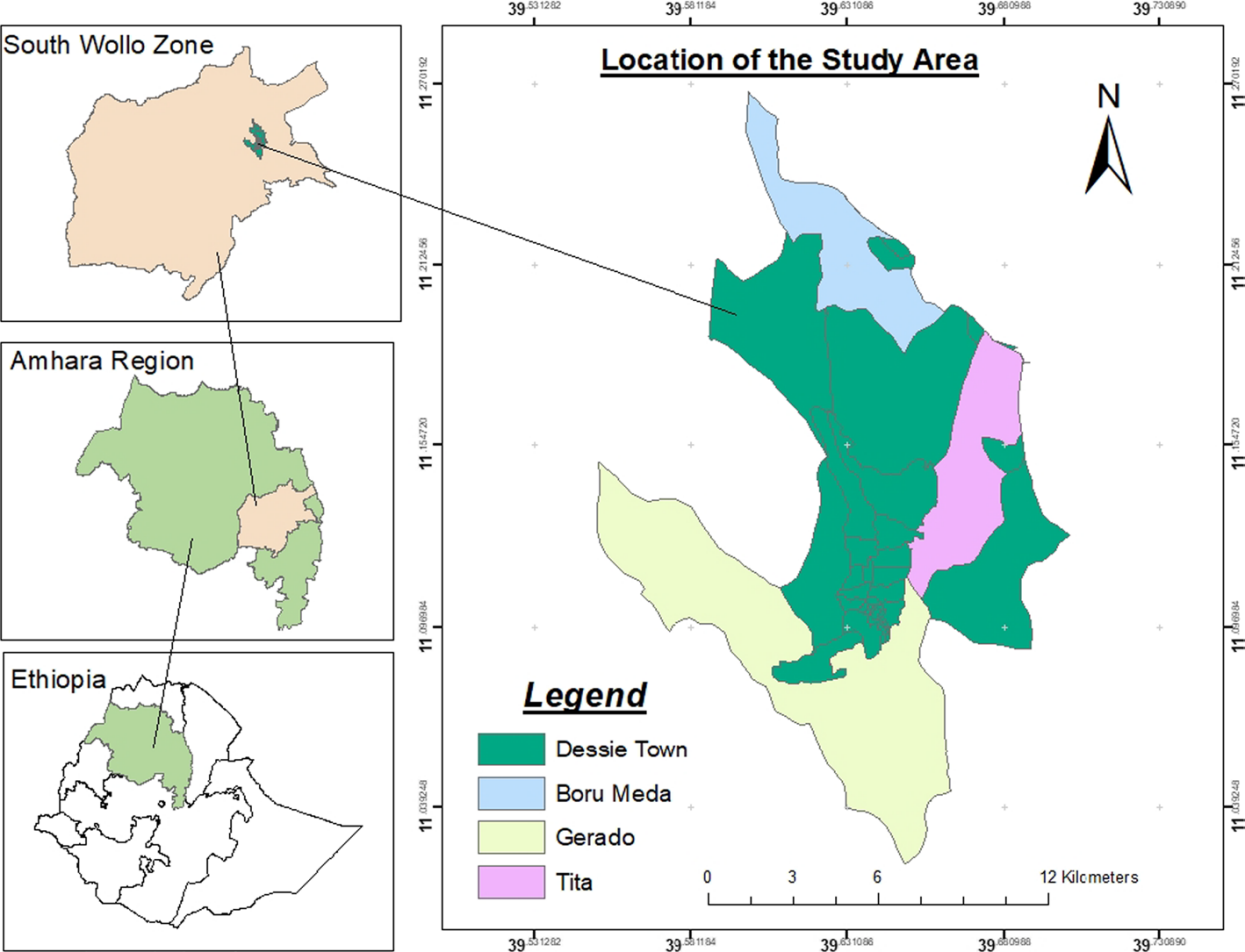
Map of the study area

The study was conducted particularly in the Dessie Zuria district of the Amhara region of Ethiopia (Fig. 1). Dessie Zuria is one of the districts in the Amhara Region of Ethiopia. Dessie Zuria is bordered on the south by Albuko and Were Ilu, on the southwest by Legambo, on the northwest by Tenta, on the north by Kutaber, on the northeast by Tehuledere, and the east by Kalu. It is sited at a latitude and longitude of 11°8′N 39°38′E, with an elevation between 2,470 and 2,550 m above sea level. The capital city of the district, Dessie, is located 400 km from the capital of the country, Addis Ababa; and 475 km from the capital of the Amhara Regional State, Bahir Dar. (Dessie Zuria District Finance and Economic Development Office, 2020).

### Sampling

A cross-sectional survey design was employed with both quantitative and qualitative components. A multi-stage sampling procedure was employed to select representative sample households. In the first stage, the Dessie Zuria District from the South Wollo Zone of the Amhara region was selected purposively due to its higher number of international migrants to the Middle East. In the second stage, three representative Kebeles^2^ were selected randomly from twenty-five kebeles(i.e. *BoruMeda*, *Gerado*, and *Tita*). Then, in the third stage, the households in each randomly selected Kebeles were stratified into migrant-sending and non-migrant sending households. Migrant-sending households can be defined as those that have at least one migrant member during the study period. Non–migrant-sending households are defined as households that have no migrant member in the household. Finally, a systematic random sampling technique was employed to select 346 households. The sample size for the study was determined by the formula given by Yamane (1967).^3^ Table 1 summarizes sampled respondents from each Kebele, calculated proportion to total household size.

**Table 1:** Sample respondents of the study area in each Kebele

Household	Total households			Sampled households			Total Sampled HHs
	BoruMeda	Gerado	Tita	BoruMeda	Gerado	Tita	
Migrant-sending HHs	350	520	458	45	68	60	173
Non-migrant sending HHs	520	455	262	73	64	36	173
Total	870	975	720	118	132	96	346

### Sources of data and method of data collection

The study used data generated from both primary and secondary sources. A semi-structured questionnaire was used to collect the required primary data. Primary data were collected from 346 randomly selected household heads of both migrant-sending and non-migrant sending households. In addition, focus group discussions (FGDs) were conducted with different stakeholders who have direct and indirect contact and role in migration. These include community leaders and migrant-sending families in each kebeles. Six focus group discussions were conducted (two from each of the three Kebeles), each comprising three male and three female-headed households. Moreover, key informant interviews (KIIs)were conducted with experts who work in the migration office, and office of labor and social affairs at the district and Kebelea dministrative bodies of the study area. Secondary data were collected from journals and published and unpublished office reports**.**

### Methods of data analysis

Both descriptive statistics and econometric models were employed to analyse the data. Descriptive statistics such as mean, standard deviation, percentage, and inferential statistics such as t-test and chi-square test were used to compare the mean difference among the treatment and control groups concerning the various socio-economic variables. The logit model was used to identify factors determining international migration. The impact of international migration on households’ income was analysed by the PSM model. The precise econometric model to analyse the impact or quantify the effect of a certain treatment is the Double Difference or Difference-In-Difference (DID) method. However, due to a lack of baseline data, the impact of remittance on households' income was analysed using a “with–without” comparison with Propensity Score Matching (PSM) with the view of comparing the comparable groups using the counterfactual data.

#### Specifications of the logit model (for determinants of international migration)

According to Gujarati (2004), the mathematical formula for the logit model taking the natural logarithm is as follows:

1 $${l}_{i}={l}_{i}\left[\frac{{p}_{i}}{1-{p}_{i}}\right]={z}_{i}={\beta }_{0}+{\beta }_{1}{x}_{1}+{\beta }_{2}{x}_{2}+\cdots+{\beta }_{n}{x}_{n}$$

where β_0_ is intercept β_1_, β_2_ … ${\beta }_{n}$ are the slopes of the equation in the model${l}_{i}$ = is the odd ratio which is not only linear in xi but also linear in parameters, Xi = pre-intervention characteristics of an individual in the study area, International labour migration is the dependent variable. A household is considered migrant-sending if it has at least one migrant member for the past year.

#### Specifications of the PSM model (for impact of international migration)

PSM matches each participant household (migrant-sending) with a non-participant household (non-migrant sending) that has almost the same likelihood of participating in the program. This study also applies a propensity score matching technique to analyse the average treatment effect on the treated (migrant-sending households). The propensity score matching approach aims to only compare households that lie in the common support and exclude others from the analysis. Unlike econometric regression methods, PSM compares observations and does not rely on parametric assumptions to identify impacts on the program. Even though PSM has many advantages, it has its limitations such as it requires large samples, group overlap, and hidden bias may occur because it’s matching only controls for observed variables. It attempts to estimate the average impact of treatment on treated/ATT (Haile, 2008).

According to Caliendo and Kopeinig (2005), there are six steps in implementing PSM. These include first the estimation of the propensity scores (by using either logit or probit models to predict the probability of participation of the household). Secondly, choosing a matching algorithm/matching estimator which best estimates the p-score. Thirdly, checking on common support conditions where the balancing score has positive density for both treatment and comparison units ensures that any combination of characteristics observed in the treatment group can also be observed among the control group. Fourthly, testing the matching quality by using the t-test is used to examine whether the mean of each covariate differs between the treatment and the control group (Rosenbaum & Rubin, 1983, 1985). The distribution of the relevant variables in both the treatment and the control group should have similar household characteristics after matching. Before matching, differences are expected but after matching the covariates should be balanced in both groups and hence no significant differences should be found. Fifthly, an estimation of the treatment effect on the treated (ATT) was conducted. To estimate the effect of international migration on a given outcome (Y) is specified as:

2 $${\tau }_{ATT=}{Y}_{i\left({D}_{i }=1\right) -\left({D}_{i }=0\right)}$$

where τi is the treatment effect (effect due to international migration), Yi is the outcome on household i, Di is whether household i have got the treatment or not (i.e., whether a household participated in international migration or not). But, Yi (Di = 1) and Yi (Di = 0) cannot be observed for the same household at the same time. Depending on the position of the household in the treatment (Participation in international migration), either Yi (Di = 1) or Yi (Di = 0) is an unobserved outcome (counterfactual outcome). Due to this fact, estimating individual treatment effect τi is not possible and one has to shift to estimate the average treatment effects of the population to the individual one. Two treatment effects are most frequently estimated in empirical studies (Caliendo & Kopeinig, 2005). One is the (population) Average Treatment Effect (ATE) which is simply the difference between the expected outcomes.

3 $$\Delta {Y}_{ATE}=E\left(\Delta {Y}_{1}\right) -E\left(\Delta {Y}_{0}\right)$$

The Average Treatment Effect (ATE) answers the question of what would be the effect if households in the population were randomly assigned to treatment. However, this estimate might not be of importance to policymakers because it includes the effect for which the intervention was never intended. So, the most important evaluation parameter is the so-called Average Treatment Effect on the Treated (ATT), which concentrates solely on the effects on those for whom the program/interventions are introduced(in this case for households who have international migrants). In the sense that this parameter focuses directly on those households who have migrants, it determines the realized impact of international migration and helps to decide whether participation in international migration is successful or not. It is given by:

4 $${\tau }_{ATT}=E\left(\tau /D =1\right)=E\left({Y}_{1}/D =1\right)-E\left({Y}_{0}/D =1\right)$$

The average treatment effect on the treated (ATT) answers the question of how much households participating in international migration benefit as compared to what they would have experienced without migrating. Data on E (Y1/D = 1) are available from migrant-sending households.

An evaluator’s classic problem is to find E (Y0/D = 1). Therefore, the difference between E (Y1/D = 1) − E (Y0/D = 1) cannot be observed for the same household. The possible solution is to use the mean outcome of the comparison individuals, E (Y0/D = 0), as a substitute for the counterfactual mean for those being treated, E (Y0/D = 1) after correcting the difference between treated and untreated households arising from the selection effect. Thus, by rearranging, and subtracting E (Y0/D = 0) from both sides of Eq. (2), one can get the following specification for ATT.

5 $$E\left({Y}_{1}/D =1\right)-E\left({Y}_{0}/D =1\right)={\tau }_{ATT}+E\left({Y}_{0}/D =1\right)-E\left({Y}_{0}/D =1\right)$$

Both terms on the left-hand side are observables and ATT can be identified, if and only if E (Y_0_/D = 1)-E (Y_0_/D = 0) = 0. i.e., when there is no self-selection bias. This condition can be ensured only in social experiments where treatments are assigned to units randomly (i.e., when there is no self-selection bias) (Caliendo & Kopeinig, 2005). In non-experimental studies, one has to introduce some identifying assumptions to solve the selection problem. The following are three assumptions to solve the selection problem.

**A. Conditional Independence Assumption (CIA):** given a set of observable covariates (X) that are not affected by treatment, potential outcomes are independent of treatment assignment. This assumption implies that the selection is solely based on observable characteristics, and variables that influence treatment assignment and potential outcomes are simultaneously observed (Caliendo & Kopeinig, 2005).

**B. Common Support:** this assumption rules out the perfect predictability of D given X. That is**:** 0 < P (D = 1| X) < 1. This assumption ensures that the same X values have a positive probability of being both migrants and non-migrants. Given the above two assumptions, the PSM estimators of ATT can be written as:

6 $${\tau }_{ATT}=E\left({Y}_{1}-{Y}_{0}/D =0, P(X)\right)=E\left({Y}_{1}/D =1,P(X)\right)-E\left({Y}_{0}/D =0,P(X)\right)$$

where P(X) is the propensity score computed on the covariates X. Equation (5) is explained as; the PSM estimator is the mean difference in outcomes over the common support, appropriately weighted by the propensity score distribution of participants.

Estimation of standard error testing: The statistical significance of treatment effects and computing their standard errors is not a straightforward thing to do. The problem is that the estimated variance of the treatment effect should also include the variance due to the estimation of the propensity score, the imputation of the common support, and possibly also the order in which treated individuals are matched.

**C. Bootstrapping:** one way to deal with this problem is to use bootstrapping. This method is a popular way to estimate standard errors in case analytical estimates are biased or unavailable. Each bootstrap draw includes the re-estimation of the results, including the first steps of the estimation (propensity score, common support). Bootstrap standard errors attempted to incorporate all sources of error that could influence the estimates. Repeating the bootstrapping N times leads to N bootstrap samples and in case N estimated average treatment effects and Bootstrapping estimate of standard errors is invalid for nearest neighbour matching selection. Thus, calculating analytical standard error is applicable here.

In the sixth step, sensitivity analysis was conducted. Unobserved variables which affect the assignment into treatment and the outcome variable simultaneously, a hidden bias might arise. Matching estimators are not robust against hidden bias arising from unobserved variables which affect the assignment into treatment and the outcome variable simultaneously. Therefore, the sensitivity of estimated treatment effects concerning unobserved covariates and Rosenbaum bounds should be tested and calculated. In cases where results are very sensitive, identifying assumptions and alternative estimators were considered. Common support i.e. the sensitivity of estimated treatment effects for the common support problem was also tested and Lechner-bounds were calculated (Caliendo & Kopeinig, 2005, 2008).

Before the actual commencement of the data analysis, a multicollinearity diagnosis test was carried out to filter independent variables that are dependent on each other. To this effect, the presence of high collinearity is tested using the Variance of Inflation Factor (VIF) for continuous explanatory variables. Furthermore, to test the association between dummy variables contingency coefficient was calculated. A heteroskedasticity diagnosis test was also carried out to detect whether the variance of the error term is different for different values of the independent variable or to detect if the error variance is constant or not (“Appendix 2”). The results show that there are no multicollinearity and heteroskedasticity issues in the models (“Appendices 2 and 3”).

#### Measurement of Variables

**Participation in International Migration (Migration Status):** is the dependent variable of the model and a dummy variable representing the migration status of a household. Migrant-sending households were represented by 1 and non-migrant households were represented by 0.

**Household’s Annual Income:** is an outcome variable that is measured in Ethiopian Birr (ETB). It is the annual income of a household from crop production, livestock production, and off/non-farm income. The hypothesis is that a migrant-sending household improves its household income more than the non-migrants through remittance.

#### Independent variables

The independent variables for the study are identified and listed based on previous theoretical and empirical works, and personal observation. The following explanatory variables (Table 2) are hypothesized to be the determinant factors of international labour migration.

**Table 2:** Summary of hypothesized explanatory variables

Variables	Variable type	Measurement	Expected sign
Sex of household head	Dummy	0 = if female; 1 = if male	−
Age of respondent	Continuous	Measured in years	−
Marital status of the HH	Categorical	Married (reference category) If single = 1; 0 = otherwise If divorced = 1; 0 = otherwise If widowed = 1; 0 = otherwise	−
Family size	Continuous	Measured in Numbers	+
Educational status of household head	Categorical	Illiterate (reference category) If only write and read = 1; 0 = otherwise If from grade 1 to 8 = 1; 0 = otherwise If from grade 9 to 10 = 1; 0 = otherwise If from grade 11 and above = 1; 0 = otherwise	+
Employment status of household head	Dummy	0 = if unemployed 1 = if employed	−
Use of credit	Dummy	0 = if has no credit access 1 = if has credit access	−
Number of livestock	Continuous	Measured in TLU^a^	−
Engagement in non/off-farm activities	Dummy	0 = if not engaged in non/off-farm activities 1 = if engaged in non/off-farm activities	−
Member of cooperatives	Dummy	0 = if not a member 1 = if member	−
Farmland size of households in hectare	Continuous	Measured in hectare	−
Network with migrants	Dummy	0 = if has no network 1 = if has network	+
Peer/family influence	Dummy	0 = if no peer/peer influence 1 = if influenced by peer/family	+

^a^TLU is Tropical Livestock Unit. Please refer to “Appendix 1” for TLU conversion factors

## Results

### Determinants of International Migration

#### Sex of the household head

The sample households were composed of 173 migrant-sending households and 173 non-migrant sending households. The survey result showed that out of the total 346 sampled households, 65.9% and 34.1% were male-headed and female-headed households, respectively. The result also indicated that 53.76% and 46.24% of migrant-sending households and 78.03% and 21.97% of non-migrant sending households were male-headed and female-headed households, respectively. The Chi-square test indicates that there is a statistically significant difference between heads of migrant-sending and non-migrant households in terms of their sex (Table 3). In addition, the logistic regression result (Table 4) shows that being a male-headed household head influences the probability of international migration negatively and significantly. The marginal effect estimation shows that being in a male-headed household decreases the probability of international migration by a unit of 0.1896, keeping other things constant. This indicates that members of female-headed households are more migratory than their male-headed counterparts.

**Table 3:** Descriptive statistics of sample households (for dummy and categorical variables)

Variables	Categories	Migrant sending HHs		Non-migrant sending HHs		Total		χ2
		Freq	%	Freq	%	Freq	%	
Sex of household head	Male	93	53.76	135	78.03	228	65.9	22.67***
	Female	80	46.24	38	21.97	118	34.1	
Marital status	Married	112	64.74	136	78.61	248	71.68	13.35***
	Single	1	0.58	4	2.31	5	1.45	
	Divorced	47	27.17	22	12.72	69	19.94	
	Widowed	13	7.51	11	6.36	24	6.94	
Education level	Illiterate	82	47.4	70	40.46	152	43.93	3.81
	Can only read and write	29	16.76	42	24.28	71	20.52	
	Grades 1 to 8	53	30.64	50	28.9	103	29.77	
	Grades 9 to 10	8	4.62	9	5.2	17	4.91	
	Grade 11 and above	1	0.58	2	1.16	3	0.87	
Employment status	Employed	86	49.71	102	58.96	188	54.34	2.98*
	Unemployed	87	50.29	71	41.04	158	45.66	
Use of credit	Access to credit	53	30.64	68	39.31	121	34.97	2.86*
	No access to credit	120	69.36	105	60.69	225	65.03	
Engagement in off/non-farm activities	Participant	63	36.42	73	42.2	136	39.31	1.21
	Non-participant	110	63.58	100	57.8	210	60.69	
Membership to cooperatives	Member	88	50.87	115	66.67	203	58.67	8.64***
	Non-member	85	49.13	58	33.53	143	41.33	
Network with migrants/returnees	Have network	115	66.47	76	43.93	191	55.2	17.78***
	Have no network	58	33.53	97	56.07	155	44.8	
Peer/family influence	Influenced	130	75.14	91	52.6	221	63.87	19.05***
	Not influenced	43	24.86	82	47.4	125	36.13	

^***^, and * indicate the level of significance at 1% and 10%, respectively

Source: own survey result, 2021

**Table 4:** Descriptive statistics of sampled households (for continuous variables)

Variables	Migrant sending HH		Non-migrant HH		Combined		t-value
	Mean	SD	Mean	SD	Mean	SD	
Age	51.57	10.78	50.66	8.78	51.11	9.83	− 0.858
Family size	5.486	1.681	5	1.34	5.242	1.54	− 2.966***
No. livestock	3.13	2.268	3.82	2.48	3.47	2.27	2.85***
Cultivated land size	0.6778	0.429	0.915	0.49	0.796	0.479	4.776***

^***^ indicates the level of significance at 1%

Source: own survey result, 2021

#### Marital status of the household head

Out of the total sampled household heads, about 71.68% of them were married and the remaining 1.45%, 19.94%, and 6.94% were single, divorced, and widowed respectively. The result also indicates that 64.74% of migrant-sending and 78.61% of non-migrant sending households were married. The remaining 0.58%, 27.17%, and 7.51% of migrant-sending households and 2.31%, 12.72%, and 6.36% of non-migrant sending households were single, divorced, and widowed respectively. The Chi-square test shows that the marital status of household heads has a statistically significant difference between migrant-sending and non-migrant sending households, implying that more non-migrant sending household heads are married and more migrant-sending household heads are divorced and widowed. The logistic regression result shows that the marital status of household heads has no significant influence in determining the international migration of members.

#### Education level of household head

Education is one of the important institutional variables. Education plays a great role in any decision in the lives of an individual and households by enhancing their capacity to acquire information about the world around them and process that information to reach a certain decision in their life. Education also influences the information-seeking behaviour of an individual. In addition to this, education increases individuals’ capacity of analysing their life situations and solving their problems (Haile, 2015). According to the result (Table 3), the total respondents' education level revealed that 43.93%, 20.52%, 29.77%, 4.91%, and 0.87% were illiterate, read and write, elementary school, high school, and preparatory and above respectively. The result also indicates that 47.4% and 40.46% of migrant-sending and non-migrant household heads were illiterate, respectively. The result indicates that there is a high illiteracy rate in the study area. The chi-square test indicated that there was no significant statistical association between migrant-sending and non-migrant households in terms of the education level of the household head. The logistic regression result also indicated that there is no significant relationship between the education level of the household head and the probability of migration (Table 5).

**Table 5:** Descriptive results of outcome variable

Variable	Migrant HH	Non-migrant HH	Combined Mean	Mean difference	t-value
Households’ annual Income	34,977.68	21,883.59	28,430.64	13,094.09	14.19***

^***^ indicates the level of significance at 1%

Source: own survey result, 2021

#### Employment status of household head

The result in Table 3 showed that 54.34% of the total sample households were employed, while 45.66% were unemployed. Out of this, 49.71%and 58.96% of the migrant-sending and non-migrant household heads were employed. The Chi-square test result indicates that there is a statistically significant difference between migrant-sending and non-migrant households in terms of employment status implying that household heads without jobs have a higher tendency to send out more migrants than employed household heads. However, the logistic regression result shows that the employment status of household heads has no significant effect on the migration status of households.

#### Access to credit

Credit is an important source of finance for poor households to buy inputs for agricultural production and improve their livelihoods. The use of credit services is one of the alternative strategies to diversify rural households’ livelihood in the Amhara region of Ethiopia in general and in the study area in particular. The use of credit also promotes households to diversify their income sources, which can reduce the migration of households. However, the majority of the sampled households could not obtain credit services. From the total respondents, only 39.31% and 30.64% of migrant-sending and non-migrant households had access to credit from ACSI^4^ and other cooperatives. This result indicated that most of the respondents had no access to credit due to a lack of collateral and high-interest rates. The chi-square analysis result revealed that there exists a statistically significant difference between migrant-sending and non-migrant households in terms of access to credit. Discussion held with key informants and focus group discussants revealed that access and use of credit create an opportunity to participate in different non-farm activities and diversify their livelihood, thereby reducing the probability of international migration. However, the logistic regression result shows that access to credit has no significant influence on the migration status of households (Table 6).

**Table 6:** Logit regression results for determinants of international migration

Variables	Coefficients	SE	Z value	*p* > z	dy/dx
Constant	0.419	0.419	0.45	0.656	
Sex	− 0.7708373	− 0.265555	− 2.90	0.004***	− 0.1896
Age	.01196	.0136586	0.88	0.381	0.00299
Maritalstatus	.1656407	.1236445	1.34	0.180	0.0414
Familysize	.2056094	0.0886297	2.32	0.020**	0.0514
Edustatus	− .0975925	.1268917	− 0.77	0.442	− 0.0243
Empstatus	− .2984862	.2494483	− 1.20	0.231	− 0.07447
Nooflivestock	− .1196206	.0557353	− 2.15	0.032**	− 0.0299
Useofcredit	.2699603	.264687	1.02	0.308	0.0673
Partinofnonfarac	− .4973304	.2641693	− 1.88	0.060*	− 0.1236
Coopmember	− .2520495	.2619101	− 0.96	0.336	− 0.0629
Farmlandsize	− .9516502	.2806547	− 3.39	0.001***	− 0.2379
Networwithmigrants	.593035	.2569952	2.31	0.021**	0.1472
Peerorfamlypre	.6705901	.2756585	2.43	0.015 **	0.1658
Number of obs	346				
LR chi2(16)	82.62				
Prob > chi2	0.000				
Log-likelihood	− 198.52				
Pseudo R^2^	0.17				

^***^, ** and * indicate the level of significance at 1%, 5% and 10%, respectively

Source: Own survey result, 2021

#### Engagement in non/off-farm activities

Off/non-farm activities are additional activities made by households through engagement in certain jobs besides farming to diversify income sources. Out of the total sampled households, about 39.31% of them replied that their family members are engaged in off/non-farm activities. The result also indicates that 36.42% of migrant-sending households and 42.2% of non-migrant households engaged in non-farm activities and got additional income. The remaining 63.58% of migrants and 57.8% of non-migrant households did not participate in non/off-farm activities. The result indicates that most households do not participate in non/off-farm activities. Engaging in non/off-farm activities helps households to diversify their livelihoods and to increase income, which reduces international migration. The Chi-square test shows no statistically significant difference between migrant-sending and non-migrant households concerning the participation of off/non-farm activity of household (Table 3). The logit regression result indicates that engagement in off/non-farm activities affected international migration negatively and significantly. Being a participant in off/non-farm activities decreased the probability of the households having an international migrant by a unit of 0.1236, keeping other variables constant. This is because households who have engaged in non/off-farm activities would have better income and capability to purchase farm inputs, additional foods, and health security than those with no engagement in non/off-farm activities.

#### Membership in local cooperatives

Among the sampled households, 58.67% are cooperative members. The result also indicates that 50.87% and 66.67% of migrant-sending households and non-migrant sending households are members of cooperatives, respectively. Although the Chi-square test shows a significant difference between migrant-sending and non-migrant households in terms of membership to cooperatives, the logistic regression result shows no significant association between membership to cooperatives and the probability of international migration.

#### Network with migrants/returnees in the destination region

Information exchange and networking with migrants are key determinant factors for international migration in the study area. More than half of the migrants have early information about the destination area and they also have a network with migrants or returnees. The result indicates that 66.47% of migrants have a network with other migrants or returnees. The Chi-square test shows a statistically significant difference between migrant-sending and non-migrant households in terms of network with migrants and returnees. The logistic regression result indicates that having a network with migrants or returnees affects international migration positively and significantly. Having a network with international migrants or returnees increases the probability of households sending migrants internationally by a unit of 0.1472. The findings from FGD indicate that migrants and returnees often provide exaggerated information about the destination area which induces more potential migrants from the study area. A community elder at GeradoKebele explained the following during the FGD.

*The majority of the youth from our area ask returnees and migrant-sending families about the kind of job available in the destination region, payment schemes, and lifestyles before the migration starts. However, some migrants and returnees at origin often mislead them and hardly tell them the reality on the ground.*

#### Peer/family influence

Pressure from peers or family has played a great role in inducing individuals for international migration to the study area. Of the total respondents, 63.87% faced peer/family pressure. In addition, 75.14%of migrants have been influenced either by their families or friends to emigrate to other countries predominantly for economic reasons. The Chi-square test result shows the existence of a significant difference between migrant-sending and non-migrant households concerning peer/family pressure for emigration. The logistic regression results show that peer or family pressure affects international migration positively and significantly. The model result indicates that the presence of peer/family pressure has increased the probability of having an international migrant by a unit of 0.1658, other things being constant. A woman discussant from TitaKebele expressed her views about the impact of peer/family influence on migration as:

*My neighbour always talks about her son who lives in Saudi Arabia and she tells me the amount of money and the type of materials he sends to his family. When I visit their house, I am inspired by the blankets and the carpets they have. Their house does not look like a farmer's house. It was beautifully made. I became eager to make my house like that and immediately I decided to send my daughter to Saudi Arabia.*

#### Age of the household head

The age of the household head could influence whether the household members are to be a migrant or not. The mean age of migrant-sending and non-migrant households at the time of the survey was 51.57 and 50.66 years, respectively with no statistically significant mean difference (Table 4). The logistic regression result shows no significant association between the age of the household head and the probability of sending a family member to an international destination.

#### Family size

Family can be defined as the number of people living together and utilizing resources of the family together. Family size is an important determinant factor of migration. Family is also the main labour source in the study area. The result indicates that the mean family size in the study area is 5.2 with a standard deviation of 1.54. The minimum family size is 2 and the maximum is 10. The mean family size of migrant-sending and non-migrant sending households is 5.5 and 5, respectively with a statistically significant mean difference between the two groups (Table 4). As shown in Table 4, there was a significant difference between migrant-sending and non-migrant sending households. The logistic regression result shows that a bigger family size induces international migration positively and significantly. The result shows that when household size increased by one member, the probability of households having an international migrant increased by a unit of 0.05, with other variables remaining constant. This implies that those households with more family members are more likely to migrate internationally. When family size increases, the per capita income of a household decreases, and normally the household look for alternative livelihoods to enhance the family’s source of income. Therefore, members of a family could choose migration as a livelihood diversification strategy. A male discussant at Gerado Kebele expressed his views regarding family size as:

*Large family size is a key push factor for international migration in our area. I observed a direct and clear association between big family size and migration.*

#### Number of livestock (in TLU)

Ownership of larger livestock is the symbol of wealth and plays a crucial role in determining migration decisions in the study area. Livestock provides milk, meat, traction power, and transport. Livestock species mostly owned by the sampled households include cattle, sheep and goats, donkeys, horses, mules, and poultry. The result indicates that the respondents in the study area have a minimum of 0 and a maximum of 9.96 livestock in TLU. The mean livestock population owned by the sample respondents was 3.47 in TLU with a standard deviation of 2.27. The mean livestock holding for migrant-sending and non-migrant households was 3.13 and 3.82 TLUs, respectively with a statistically significant mean difference. The logistic regression result indicates that larger ownership of livestock discourages significantly the motive to migrate internationally. A unit increase in households’ livestock by one TLU has decreased the probability of having an international migrant by a unit of 0.0299, keeping other variables constant. This implies that households with smaller livestock numbers have a higher probability of international migration. The possible justification is that owning more livestock may help to timely tillage of land, diversification of risks, and additional income from the sale of livestock and their by-products. The income obtained from livestock helps also in purchasing farm inputs and also enhancing the food security of the household. In addition, livestock particularly cattle serve households as draught power for the cultivation of land. In addition, animals such as donkeys, mules, and horses serve as a means of transporting human beings and farm products. The manure from the animals also fertilizes farmlands and improves yield per plot of land.

#### Land size

Land is one of the main productive assets in agrarian countries like Ethiopia. Cultivable land is the most important but scarce resource for production. In the study area, the average landholding size of the sample households is 0.796 hectares with a standard deviation of 0.479. The average land size for migrant-sending households was 0.678 ha with a standard deviation of 0.429 while for non-migrants was 0.915 ha with a standard deviation of 0.49. The t-test result shows that the mean difference between migrant-sending households and non-migrant households concerning land size was statistically significant (Table 4). The logistic regression result indicates that land size affected the probability of migration negatively and significantly. A hectare increase in the size of land decreased the probability of migration by a unit of 0.238, keeping other variables constant (Table 6). Households that have a larger size of land have a higher probability for crop diversification while those who have smaller land are poorer and more likely to migrate. KIIs and FGDs conducted in the study area confirm that land is an important indicator of wealth in the study area. Households owning a larger size of land can afford to produce enough for both household consumption and sale, which potentially leads to a lower inclination towards international migration. A key informant in the study area explained his views about land ownership in the locality as:

*The average plot of cultivable land per family is very small with low productivity. The majority of the land is used for food production and cash crop production in the area is insignificant. Moreover, most of the grandsons and granddaughters who are becoming householders do not have their plots of land and thus, they are dependent on their parent's land.*

### The impact of international migration on the income of households staying behind

**Fig. 2:**
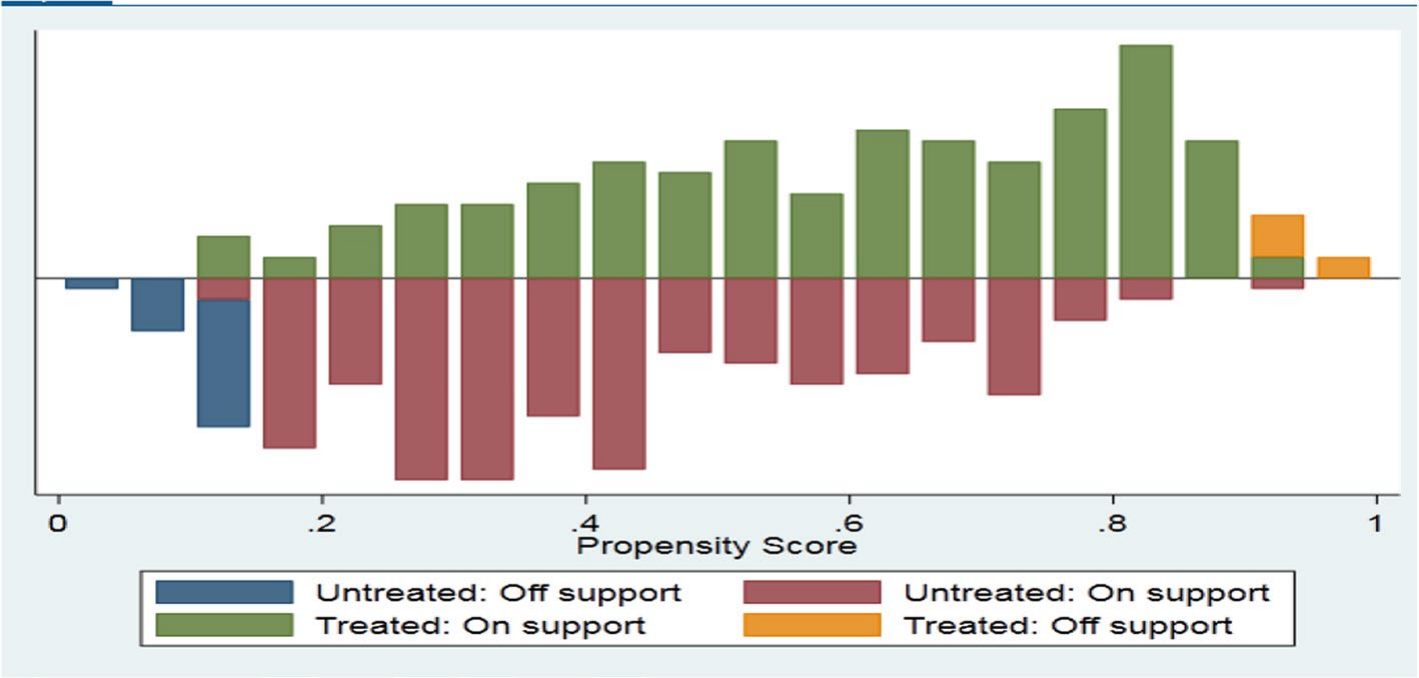
Distribution of propensity score of the treated and untreated households before matching

The impact of international migration on households’ income was analysed using a propensity score matching model. The estimation of propensity scores, matching methods, common support region, matching quality, treatment effect, and sensitivity analysis of the sample household are presented and discussed. The p-score graph was used to show the distribution of the households concerning the estimated propensity scores. Figure 2 shows the distribution of propensity scores of both treatment and control observations before a common support condition is imposed. The figure revealed that there appeared unmatched observations in both the treated and control groups before a common support condition is imposed.

The combined mean of the annual income of the sampled households was ETB 28,430.64. The average annual income of the migrant-sending sample households and non-migrant households was ETB 34,977.68 and 21,883.59 respectively. The mean difference between the income of migrant-sending and non-migrant sending households was ETB 13,094.09. The households' annual income between the migrant-sending and non-migrant households was significantly different at the 1% level (Table 5).

#### Matching participant and comparison households

The estimated propensity scores vary between a maximum of the minimums (0.135) and the minimum of the maximums (0.91) of the total household as the result indicated in Table 7. For this study, the common support region would then lie between 0.135 and 0.91, which means that households whose estimated propensity scores are less than 0.135 and greater than 0.91 are discarded from the analysis. As a result of this restriction, 24 households (18 non-migrant households and 6 migrant-sending households) were discarded from the exercise.

**Table 7:** Distribution of sample households in estimated propensity score matching

Group	Observation	Mean	SD	Min	Max
Treated	173	0.60	0.21	0.135	0.978
Controlled	173	0.39	0.20	0.046	0.91
Total	346	0.50	0.234	0.135	0.91

Source: Own survey result, 2021

**Fig. 3:**
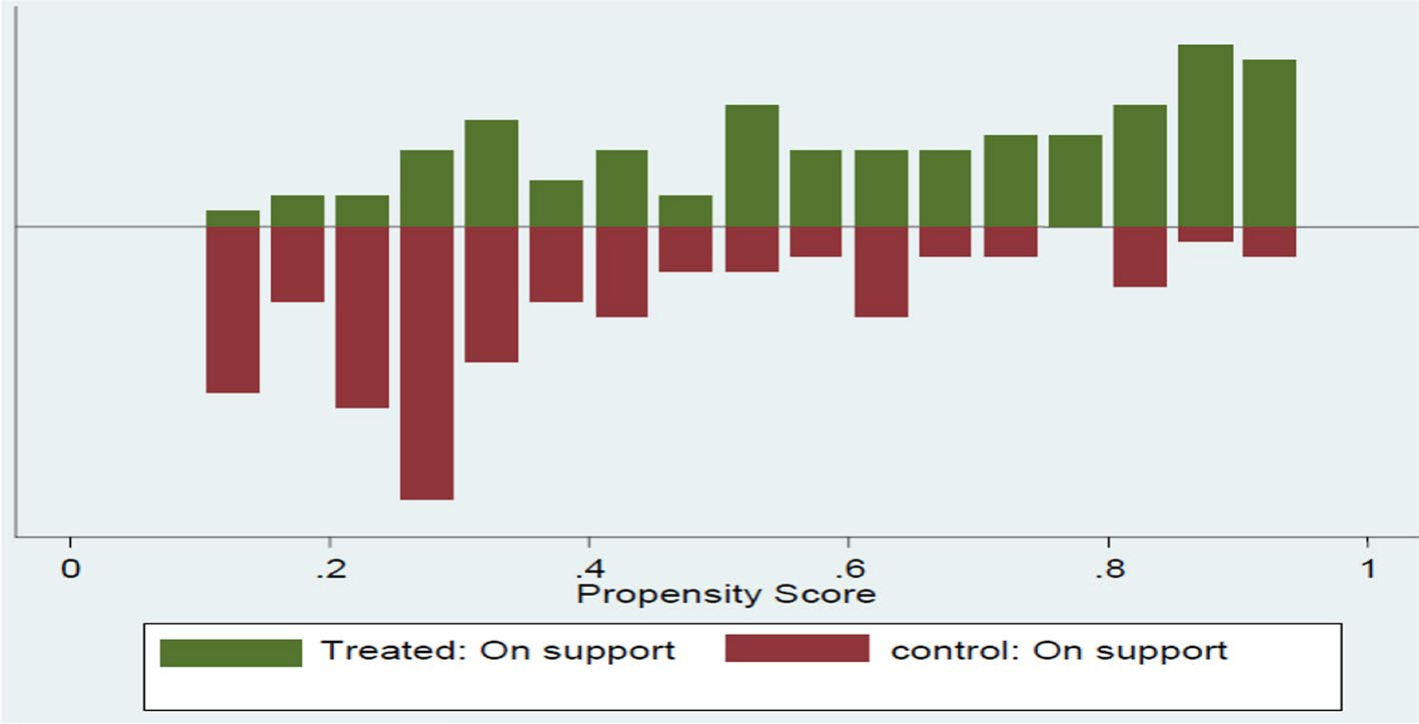
Distribution of propensity score of the treated and untreated households after matching

In addition to this, the study also used the p-score graph to test the plausibility of the overlap assumption. Figure 3 shows the distribution of propensity scores of treated and control households after common support. The figure revealed that there is no unmatched observation in both the treated and control groups after a common support condition is imposed.

#### Choice of matching algorithm

The estimated result of tests of matching quality was based on the above-mentioned performance criteria as indicated in Table 8. According to the results, it has been found that radius matching with a bandwidth of 0.1 was found to be the best estimator for the data with a low pseudo-R^2^ value of 0.011 and a larger matched sample size as compared to other alternative matching estimators. The estimation results and discussion are the direct outcomes of the radius matching algorithm based on a bandwidth of 0.1.

**Table 8:** Performance criterion of the different matching algorithm

Performance criteria			
Matching estimators	Balancing test	Pseudo R^2^	Matched sample size
Kernel matching			
Band width 0.01	11	0.028	292
Band width 0.1	13	0.013	322
Band width 0.25	13	0.014	322
Band width 0.5	9	0.052	322
Calipers matching			
Band width 0.01	10	0.046	292
Band width 0.1	7	0.066	322
Band width 0.25	7	0.066	322
Band width 0.5	7	0.066	322
Radius Calipers			
Band width 0.01	11	0.023	292
Band width 0.1	13	0.011	322
Band width 0.25	13	0.022	322
Band width 0.5	9	0.086	322
Nearest neighbor			
NN 1	7	0.066	322
NN 2	7	0.06	322
NN 3	10	0.04	322
NN 4	9	0.04	322
NN 5	11	0.03	322

Source: own survey result, 2021

#### Testing the balance of propensity score and covariates

The balancing powers of the estimations and covariates by applying the selected matching algorithm are ascertained by considering different test methods such as equality of means using the t-test and chi-square test for joint significance of the variables used (Alemu, 2010).

As indicated in Table 9, the t-values showed that before matching some of the chosen variables exhibited statistically significant differences while after matching all of the covariates were balanced.

**Table 9:** Propensity score and covariate balancing

Variables	Sample	Mean		Test	
		Treated	Control	t-value	*p* > t
Sex	Unmatched	0.5376	0.78	4.9130	0.000***
	Matched	0.55689	0.554	0.06	0.954
Age	Unmatched	51.57	50.66	− 0.8582	0.3914
	Matched	51.335	51.359	− 0.02	0.982
Marital status	Unmatched	1.775	1.468	− 2.803	0.005***
	Matched	1.7246	1.6303	0.82	0.410
Family size	Unmatched	5.48	5	− 2.966	0.003***
	Matched	5.4192	5.5727	− 0.87	0.383
Education status	Unmatched	1.942	2.023	0.7469	0.4556
	Matched	1.9641	1.8884	0.69	0.493
Livestock	Unmatched	3.129	3.81	2.848	0.004***
	Matched	3.187	3.1288	0.25	0.801
Access to credit	Unmatched	0.393	0.31	− 1.693	0.0913*
	Matched	0.38323	0.42048	− 0.69	0.489
Engagement in off/non-farm activities	Unmatched	0.364	0.42	1.099	0.272
	Matched	0.35329	0.36386	− 0.20	0.841
Cooperative membership	Unmatched	0.5086	0.664	2.9768	0.003***
	Matched	0.52695	0.57295	− 0.84	0.400
Land size	Unmatched	0.6778	0.91	4.77	0.000***
	Matched	0.69611	0.74334	− 0.90	0.366
Network	Unmatched	0.6647	0.439	− 4.31	0.000***
	Matched	0.6587	0.60493	1.02	0.310
Peer/family pressure	Unmatched	0.7514	0.526	− 4.477	0.000***
	Matched	0.74251	0.70008	0.86	0.389
Employment-	Unmatched	0.497	0.589	1.72	0.085*
	Matched	0.50898	0.54253	− 0.61	0.541

*** and * indicates the level of significance at 1% and 10%, respectively

Source: own survey result, 2021

Similarly after matching the low pseudo-R^2^ and the insignificant likelihood ratio tests support the hypothesis that both groups have the same distribution in covariates *X* after matching (Table 10). The chi-square test for joint significance for the radius 0.1 bandwidth matching algorithm is shown in Table 10. These results clearly show that the matching procedure can balance the two groups.

**Table 10:** Chi-square for the joint significance of variables

Sample	Pseudo R^2^	LR Chi^2^	P > chi^2^
Unmatched	0.1722	82.62	0.0000
Matched	0.011	4.9	0.977

Source: own survey result, 2021

#### Estimation of treatment effect on the treated

The impact of migration on the outcome variable (income of the households) for migrant-sending households was evaluated using the PSM model after the pre-migration differences were controlled. The estimation result presented in Table 11 provides supportive evidence of a statistically significant effect of international migration on the outcome variable, i.e. household income. A positive value of the average treatment effect on the treated (ATT), i.e. the difference between the treated and the control indicates that the income of households has been improved as a result of international migration in the study area. As indicated in Table 11, the income of migrant-sending households increased by ETB 13,079.51, which was significant at a 1% level of significance.

**Table 11:** ATT for total annual income due to international migration

Outcome variable	Treated	Control	Difference	S.E	t-value
Households’ income	34,977.2335	21,897.7203	13,079.5133	1,345.986	9.7***

^***^ indicates the level of significance at 1%

Source: own survey result, 2021

#### Sensitivity analysis

The result (Table 12) indicates that the outcome variable bears a statistical difference between migrant-sending and non-migrant sending households, while the rest of the values which correspond to each row of the significant outcome variable are p-critical values (or the upper bound of Wilcoxon significance level, Sig +) at a different critical value of e^y^ (Rosenbaum, 2002).

**Table 12:** Result of sensitivity analysis using Rosenbaum bounding approach

Outcome variable	e^y^ = 1	e^y^ = 1.5	e^y^ = 2	e^y^ = 2.5	e^y^ = 3
Households’ income	*p* < 0.00	*p* < 0.00	*p* < 0.00	*p* < 0.00	*p* < 0.00

e^y^ (Gamma) is log odds of deferential assignment due to unobserved factors where Wilcoxon significance level for outcome variable is calculated

Source: own estimation, 2021

The inference for the impact of international migration is not changing although the migrant-sending and non-sending households have been allowed to differ by up to 200% (3) (Table 12). That means for an outcome variable estimated at various levels of the critical value of e^y^, the p- critical values are significant and are considered important covariates that affected both migrant-sending households and the outcome variable. It is impossible to get the critical value e^y^ where the estimated ATT is questioned even if set the critical value largely up to 3, which is a larger value compared to the value set in different literature which is usually 2 (100%). Thus, the sensitivity analysis in Table 12 shows that impact estimates (ATT) are insensitive to unobserved selection bias and are a pure impact of international migration.

## Discussion

Households decide to emigrate taking into account several economic and non-economic factors in origin and destination areas. Lee (1966) formulated a push–pull theory of migration and argued that the determinant factors are associated with factors in the place of origin and destination, intervening obstacles, and personal factors. The decision to migrate may not necessarily depend on the assessment of the positive and negative outcomes of emigration by the migrants themselves but could be influenced by families, relatives, and friends in origin and destination places. This could eventually lead to inaccurate evaluation of factors, particularly in the destination regions resulting in imprecise decisions. While push factors in the place of origin may include deterioration of the economic conditions in the origin areas or political unrest, the pull factors in the place of destination include high job opportunities and earnings, better education, and health facilities, and the presence of migrants’ networks. Lee also argued that the intervening obstacles are those factors that are potential barriers to emigration including the cost of migration, transportation and communication problems, visa, and other emigration requirements. Personal factors are related to knowledge, awareness, and other socio-cultural factors. The early Ravenstein’s ‘Laws of migration’ (Ravenstein, 1885) also depict that migration is determined by some factors such as distance (short or long distance to destinations), location of residences (rural or urban), and migrants’ characteristics such as age and sex. Furthermore, the neoclassical theory of migration argued that migration occurs when there exists a wage differential between the origin and destination regions, where the expected earnings in the destination region are greater than the wage in the origin areas (Harris & Todaro, 1970; Todaro, 1969). The Human capital theory also argues that some variables like education and age are among the determinants for migration. While the decision to migrate declines with increased age, schooling has a positive effect on the probability of migration (Sjaastad, 1962). The New Economics of Labor Migration (NELM) explains that the decision to migrate is a collective or mutual affair where family members of migrants have stakes in the decision in developing countries. In this context, the head of the household may lead the process of decision-making in the family. Migrants and their families in their place of origin are bound together by mutually beneficial and informal contracts to provide income insurance to one another (Stark & Bloom, 1985; Taylor, 1999). Migrant networks theory argues that there exist sets of interpersonal ties that connect migrants, former migrants, and non-migrants in origin and destination areas through friendship or kinship ties. Having a network is a form of social capital that induces migration as it lowers the risks and costs of migration, and thus increases the net returns of migration (Massey et al., 1993).

Our findings corroborate the aforementioned theoretical explanations of the causes and drivers of migration. Our findings indicate that individual and household characteristics coupled with ownership of key assets and networks influence the decision to migrate. On one hand, being in a female-headed household, having a larger family size, and the presence of migrants’ network in the destination region induce migration of family members. On the other hand, households who own more assets such as arable land and livestock, and engage in non-farm businesses have a lower propensity to migrate to other areas. These imply that the decision to migrate is related mainly to improving the economic well-being of migrants and their families despite the risks they face in the course of migration. As indicated in the NELM model of migration, the decision to migrate is also a collective decision where it is not solely made by the migrant. Our findings corroborate with this model of migration that the pressure from family members or friends has significantly influenced the decision to migrate to the study area. The positive connection between peer or family pressure and the propensity of migration is consistent with the works of Esubalew (2010), Fleischer (2006), and Shishay (2019).

Our findings show that being in a female-headed household tends to influence the propensity of out-migration positively compared to the case in male-headed households. This is in line with one of Ravenstein’s laws of migration that argues females are more migratory than males (Ravenstein, 1885). Female-headed households, which are often widowed or divorced, are vulnerable to gender biases in Ethiopia. As a result, female-headed households make use of migration as a livelihood diversification strategy to achieve their life aspirations. A study conducted by Atsede and Penker (2016) shows that female-headed households in Ethiopia were 3.3 times more likely to send out prospective migrants than their male counterparts. The same study reported that households headed by females face economic disadvantages and possess less productive assets that could potentially induce the out-migration of family members to sustain their livelihoods. Ethiopia is one of the top sources of domestic workers in the Middle East, where women and adolescent girls constitute the largest share of migrant domestic workers (RMMS, 2014).

Our findings show that larger family size is one of the root causes of migration as migration serves as a diversification strategy of income sources to meet the growing demand of family members and escape poverty. Various empirical studies such as Abel et al. (2022), Czaika and Reinprecht (2020), Mendola (2008), Phuong et al. (2008), Shimelis (2010), Stark (1991), Tsegai (2007), Wondimagegnhu and Zeleke (2017), and Zhao and Zhong (2019) confirmed a positive association between family size and migration. Having a larger family size is one of the determinant variables that positively contribute to sending out migrants. Households with larger family sizes have a higher likelihood of migration. The increased demand for additional income coupled with the low level of labor productivity in the origin could be some of the reasons that induce migration for larger families. The deterioration of the agriculture sector in Ethiopia, fragmentation and shortage of land, and limited non-farm employment opportunities have been also mentioned as aggravating factors for the migration of Ethiopians (O’Neil, et al., 2016). Our findings indicate that households who are engaged in non-farm activities in addition to farming have a less likelihood of sending-out prospective migrants. Given the poor performance and productivity of the agriculture sector in the country, non-farm employment opportunities are essential in diversifying the income sources of households. In addition, households that obtain additional income from non-farm activities could be re-invested to enhance the productivity of the farming sector as they could develop the capacity to purchase agricultural inputs and utilize modern technologies. This could, in turn, increase the income obtained from farming and able to improve their livelihoods. If non-farm opportunities are lacking, individuals could still consider out-migration as a survival strategy. Our finding on the association between engagement in non-farm activities and migration is also consistent with some prior studies such as Biswas and Mallick (2021), Marchetta (2013), Melaku (2018), Phuong et al. (2008), and Tsegai (2007).

Our findings show that households that have networks in the destination regions have a higher propensity to send out prospective migrants. The availability of networks in the destination region could increase the flow of more migrants as they could have better access to information about the destination region, develop confidence and get more support that could reduce the cost of migration and integration in the destination region. A study by Simpson (2022) confirmed the role of migrants’ networks as a driver of migration. This particular study found a positive and strong effect of networks on predicting the flow of migrants. Prospective migrants are attracted by destination regions with a larger population of migrants who come from the same place of origin, speak the same language, and share a common culture (Simpson, 2022). Other empirical works such as Blumenstock et al. (2022), Eve (2022), Genicot and Dolfin (2010), Massey et al. (1993), Rainer and Siedler (2009), and Yohannes (2018) also confirmed the positive association between the presence of migrants’ networks and the likelihood of migration.

As noted in our findings, deprivation in asset ownership such as land and livestock is one of the significant push factors for migration decisions in the study area. As a result of population pressure and the establishment of new families, land fragmentation and even landlessness have become a major concern, particularly for rural households. Ownership of land per household has been diminishing from time to time, and this, in turn, has declined the production of crops as households have limited alternatives (such as access to agricultural technologies) to enhance production and productivity. Therefore, households look for alternative livelihood strategies including migration to sustain their livelihoods. Empirical studies such as Mulugeta and Makonnen (2017), Kaag et al. (2019), Schürmann et al. (2022), and Shishay (2019) depicted the relationship between land ownership and the motivation for migration, where households who own more land have less interest to migrate. Similarly, households who lack livestock resources have a higher propensity for migration than households who are better off in livestock ownership in the study area. Livestock ownership is a key indicator of wealth and serves as a diversification strategy of income sources. During crop failures, livestock serves as an insurance and coping strategy (Hänke & Barkmann, 2017).

International migration has continued to be a growing phenomenon with implications for socioeconomic development. Our findings show that migration has significantly improved the economic well-being of migrants as well as their families left behind. Remittances significantly enhance the income of households that could also improve the health and education dimensions of human development in Ethiopia. The inflow of international remittances has been significantly increasing in Ethiopia. The recent estimate from the World Bank (2021) shows that Ethiopia received almost 448 million USD as remittances in 2021, which is far higher than the amount received in 2000 (i.e. around 53 million USD). The figure would be higher if remittances received through informal channels had been counted. Yadeta and Hunegnaw (2022) estimated that the real GDP of Ethiopia would increase by 1.13% for every 1% increase in remittances in the long run. Although remittances are consumed in the short run, they could be saved and invested in the long run. A study by Zeyede (2016) found that remittances make up a significant share of households’ consumption for migrant-sending families in Ethiopia, and are also a source of savings and investment. The same study confirms the existence of a strong and positive relationship between remittances and households’ savings in Ethiopia. Several empirical studies confirmed that remittances have multiplier effects as a source of finance that could be invested in agricultural and non-agricultural businesses, and a source of insurance during natural and human-induced shocks (Atnafu et al., 2014; Ratha, 2012; Reinert, 2006; Wondimagegnhu, 2015). Despite the positive economic outcome of international migration in Ethiopia, migration has continued to be a risky investment as it could potentially expose migrants to exploitation, abuses, and violation of their rights both during their journey and in destination countries. A study on Ethiopian female domestic workers migrating to the Middle East identified that female domestic workers often work for long hours with a lower payment, and work in conditions with limited labor regulations. In addition, they experience some serious violations of their rights such as labor exploitations, sexual abuses, physical assault, trafficking, isolation and marginalization, incomplete/no wage payments, and different forms of discrimination. As a result, they have been suffering from mental and psychological trauma (McCormack et al., 2015; Mulugeta & Makonnen, 2017; O’Neil et al., 2016; Shishay, 2019). Although migrants face scary journeys and abuses in the destination region, migration has continued to be a key livelihood and poverty-escaping mechanism in Ethiopia. This makes the trade-off between the economic benefits of migration and the risks associated with it complex.

## Concluding remarks

International migration is an important livelihood strategy used as a means of generating household income and for economic growth. The study focused on analysing the impact of international migration on households’ income and identifying key determinants of international migration of Ethiopians particularly to the Middle East taking the case of the Dessie Zuria district in the Amhara region of Ethiopia.

The result of the logit model shows that among thirteen explanatory variables used in the regression analysis seven of them were found to be significant. While being a male head of household, larger livestock holding, engagement in non/off-farm activities, and ownership of larger size of farmland determine international migration negatively, family size, network with migrants/returnees, and peer/family pressure determine international migration positively. The result of the PSM model also shows that 167 migrant-sending households were matched with 155 non-migrant sending households after discarding 24 households whose values were out of the common support region by using a radius matching estimator with 0.1 bandwidths. The resulting matching passed on the processes of matching quality tests such as the t-test and chi-square test. Moreover, the computed standard error was bootstrapped to capture all sources of errors in the estimates, and finally, sensitivity analysis was made. The estimation of the impact result indicated significant differences in households’ income between treatment (migrant-sending) and control (non-migrant sending) households. The result of the Rosenbaum bounding procedure to check the hidden bias due to unobservable selection showed that the estimated ATT for the significant outcome variable was insensitive which indicates its robustness. Therefore, it is possible to conclude that international migration has a significant relationship with household income.

The study concludes that the primary motive behind migration is to move out of poverty and improve the living standards of the migrants themselves and their families through remittances although migrants pay a heavy price in the course of migration and the destination country. If economic problems in the country such as low income, smaller possession of assets such as land and livestock coupled with larger family size are not improved, migration to international destinations will continue despite the tragedies associated with it. Reducing poverty, improving the physical and financial assets of the poor, and improving alternative livelihoods such as engagement in non-farm activities, and other employment opportunities for the youth are imperative to reduce migration. If migration is inevitable, local governments should enhance the skill of migrants by preparing tailor-made training for potential migrants so that they could engage in better-paid jobs and also avoid unnecessary labour exploitations. In addition, strict legitimate actions should be taken against the brokers, human traffickers, and smugglers who mislead prospective migrants and their families through false promises, and exaggerated and deceptive information about the destinations. There is also a need to formulate a comprehensive national and international migration management policy which is imperative to better manage migration. Migration can have a positive impact on the development of policies and interventions aimed at a legal and skill-based type of migration.

## Data Availability

The datasets used and/or analyzed during the current study are available from the corresponding author upon reasonable request.

## Appendices

### Appendix 1: Conversion factors for livestock

**Table:**

No	Livestock category	Conversion factor (TLU)
1	Sheep and goat (adult)	0.13
2	Sheep and goat (young)	0.06
3	Heifer	0.75
4	Bull	0.75
5	Cow and oxen	1.0
6	Donkey (adult)	0.70
7	Donkey (young)	0.35
8	Chicken	0.013
9	Camel	1.25
10	Calves	0.25

Source: Storck et al. (1991)

### Appendix 2: Summary of Heteroskedasticity test

**Table:**

Breusch-Pagan/Cook-Weisberg test for Heteroskedasticity
H0: constant variance
Variables: fitted values of migstatus
Chi2 (1) = 0.55; Prob. > Chi2 = 0.4581

### Appendix 3: Summary of multicollinearity test

**Table:**

Variable	VIF	1/VIF
Age	1.15	0.8687
Family size	1.14	0.8750
Farmland size	1.02	0.9798
nooflivestock	1.01	0.9930
Mean VIF	1.08	

Correlation tests

**Table:**

	Sex	Marital status	Edu status	Emp status	Access to credit	Engage in non-farm activity	Cooperative member	Peers or family pressure
Sex	1.0000							
Marital status	-0.1111	1.0000						
Edu status	-0.0063	-0.0232	1.0000					
Emp status	0.0137	-0.1405	-0.1405	1.0000				
Access to credit	-0.0861	0.0048	-0.0295	-0.0761	1.0000			
Engage in non-farm activity	0.0048	0.0028	0.0963	0.0606	0.1916	1.0000		
Cooperative member	0.0400	-0.2356	-0.2356	0.0147	0.0318	-0.0368	1.0000	
Peers or family pressure	-0.1857	0.0568	0.1127	0.0473	0.0469	0.1125	-0.2036	1.0000

## Footnotes

1 ETB (Ethiopian Birr) is the currency of Ethiopia. 1 ETB is equivalent to US$ 0.022.

2 Kebele is the smallest administrative unit in Ethiopia.

3 $n={N/( 1 + N(e)}^{2}$) where n is sample size; N is total number of migrant sending and non-migrant sending households in the target Kebeles (2,565 households); e is the error of margin (5% error of margin considered).

4 ACSI refers to Amhara Credit and Saving Institution. This institution has recentry grown into a bank.
